# Factors modulating ^99m^Tc‐MAA planar lung dosimetry for ^90^Y radioembolization

**DOI:** 10.1002/acm2.13734

**Published:** 2022-07-30

**Authors:** Benjamin P. Lopez, Armeen Mahvash, James P. Long, Marnix G. E. H. Lam, S. Cheenu Kappadath

**Affiliations:** ^1^ Department of Imaging Physics University of Texas MD Anderson Cancer Center Houston Texas USA; ^2^ University of Texas MD Anderson Cancer Center UT Health Houston Graduate School of Biomedical Sciences Houston Texas USA; ^3^ Department of Interventional Radiology University of Texas MD Anderson Cancer Center Houston Texas USA; ^4^ Department of Biostatistics University of Texas MD Anderson Cancer Center Houston Texas USA; ^5^ Department of Radiology and Nuclear Medicine University Medical Center Utrecht Utrecht The Netherlands

**Keywords:** ^90^Y radioembolization, ^99m^Tc‐macro‐aggregated albumin, lung dosimetry, planar scintigraphy

## Abstract

**Purpose:**

To investigate the accuracy and biases of predicted lung shunt fraction (LSF) and lung dose (LD) calculations via ^99m^Tc‐macro‐aggregated albumin (^99m^Tc‐MAA) planar imaging for treatment planning of ^90^Y‐microsphere radioembolization.

**Methods and materials:**

LSFs in 52 planning and LDs in 44 treatment procedures were retrospectively calculated, in consecutive radioembolization patients over a 2 year interval, using ^99m^Tc‐MAA planar and SPECT/CT imaging. For each procedure, multiple planar LSFs and LDs were calculated using different: (1) contours, (2) views, (3) liver ^99m^Tc‐MAA shine‐through compensations, and (4) lung mass estimations. The accuracy of each planar‐based LSF and LD methodology was determined by calculating the median (range) absolute difference from SPECT/CT‐based LSF and LD values, which have been demonstrated in phantom and patient studies to more accurately and reliably quantify the true LSF and LD values.

**Results:**

Standard‐of‐care LSF using geometric mean of lung and liver contours had median (range) absolute over‐estimation of 4.4 percentage points (pp) (0.9 to 11.9 pp) from SPECT/CT LSF. Using anterior views only decreased LSF errors (2.4 pp median, −1.1 to +5.7 pp range). Planar LD over‐estimations decreased when using single‐view versus geometric‐mean LSF (1.3 vs. 2.6 Gy median and 7.2 vs. 18.5 Gy maximum using 1000 g lung mass) but increased when using patient‐specific versus standard‐man lung mass (2.4 vs. 1.3 Gy median and 11.8 vs. 7.2 Gy maximum using single‐view LSF).

**Conclusions:**

Calculating planar LSF from lung and liver contours of a single view and planar LD using that same LSF and 1000 g lung mass was found to improve accuracy and minimize bias in planar lung dosimetry.

## 1 INTRODUCTION

Treatment planning for ^90^Y‐microsphere transarterial radioembolization, sometimes known as selective internal radiation therapy, of liver tumors requires an estimation of the expected lung dose (LD) from microspheres shunted from the liver that become embolized in the pulmonary vasculature. In general, the LD is calculated from an estimated lung shunt fraction (LSF), the planned ^90^Y‐microsphere administered activity, and the estimated lung mass (*M*).

The instructions for use (IFU) for the two commercially available ^90^Y‐microsphere products, SIR‐Spheres (Sirtex Medical Inc., Woburn, USA) and TheraSphere (Boston Scientific, Marlborough, USA), each define procedures for estimating LSF and LDusing ^99m^Tc‐macro‐aggregated albumin (^99m^Tc‐MAA) planar scintigraphy with varying details.^1^, ^2^ The SIR‐Spheres IFU outlines administering ∼140 MBq (∼4 mCi) of ^99m^Tc‐MAA, acquiring anterior and posterior views of chest and abdomen bed positions for 0.7–1.0 million counts per image, contouring the lungs and liver in both views, and finally estimating LSF from the geometric mean of the lung and liver contour counts. The TheraSphere IFU, on the other hand, only states administering a tracer dose of ^99m^Tc‐MAA and calculating LSF using a ratio of the lung and total image counts without defining the views and bed positions imaged. Neither IFU provides detailed guidance on lung mass calculation beyond the TheraSphere IFU recommending a 1000 g *M* for all patients. Finally, both SIR‐Spheres and TheraSphere IFUs define 30 and 50 Gy LD limits for single and cumulative radioembolization treatments, but only the SIR‐Spheres IFU imposes an additional LSF maximum limit at 20%.

Although planning lung dosimetry with ^99m^Tc‐MAA planar scintigraphy is standard‐of‐care (SOC) across most clinical practices, there are various limitations affecting both the accuracy and reproducibility of the estimated planar LSF and LD values ($LSF^{\mathrm{planar}}$, $LD^{\mathrm{planar}}$).^3^ First, the diagnostic ^99m^Tc‐MAA biodistribution does not perfectly replicate the therapeutic microsphere biodistribution and will overestimate the microsphere LSF and LD primarily as a result of radiotracer degradation into free ^99m^Tc‐pertechnetate in the blood pool.^4^, ^5^ Second, planar scintigraphy does not provide patient‐specific lung mass estimates, so standard man/woman values must be applied. Finally, accurate and replicable quantification of activity distribution with planar imaging is difficult due to variable scatter and attenuation effects in the chest and abdomen, organ overlap in the projected 2D views, lack of anatomical landmarks for organ delineation, relatively low signal in the lungs, poor spatial resolution, and patient respiratory motion.^3^ In a recent study with a digital XCAT phantom, Bastiaannet et al. estimated the SOC $LSF^{\mathrm{planar}}$ geometric mean calculation overestimates LSFs in the typical clinical range of 0–20 percentage points (pp) by at least 25% and identified that the error is driven primarily because of the differences in lung and liver attenuation.^6^

Fortunately, from a patient safety standpoint, the combination of all these factors result in the predicted MAA‐based $LSF^{\mathrm{planar}}$and $LD^{\mathrm{planar}}$overestimating the true MAA‐based LSF and LD which in turn overestimates true ^90^Y‐microsphere LSF and LD values, as has been shown in both phantom and patient data.^6^, ^7^, ^8^, ^9^, ^10^, ^11^, ^12^ In fact, the incidence of radiation pneumonitis following radioembolization in current practice is extremely low, even in patients with high lung shunting estimated with ^99m^Tc‐MAA planar imaging receiving LDs above the IFU single‐ and multiple‐treatment planar‐based lung dosimetry limits.^13^, ^14^, ^15^, ^16^ Unfortunately, without established SPECT‐based LD limits and without reliable techniques to translate SPECT‐based dosimetry values to planar‐based dosimetry values, the use of pre‐therapy ^99m^Tc‐MAA SPECT imaging for lung dosimetry, if any, will vary between practices. However, the overestimation of the true LSF using SOC planar imaging has two potentially major consequences in patient care. It can result in the patient becoming ineligible to receive radioembolization altogether or receiving a lower (potentially less beneficial) tumor dose to satisfy the planar‐based LD limits.

In a 2018 survey of members of the Cardiovascular and Interventional Radiological Society of Europe (CIRSE), 82% of the 71 responding centers stated that lung shunting was the primary reason patients were excluded from radioembolization treatment.^17^ Once eligible candidates proceeded to therapy, 61% of centers reported 2%–10% of all patients required a dose reduction due to the high estimated LD. The impact of lung shunting can also be quantified by reviewing patient exclusions in the recent SARAH and DOSISPHERE clinical trials.^18^, ^19^ In these trials, 15 of 52 (29%, SARAH) and 6 of 18 (33%, DOSISPHERE) of all patients excluded after ^99m^Tc‐MAA imaging were because of high lung shunts. If lung shunts estimated with planar imaging were not used as an exclusion criterion, an additional 8% (15+174, SARAH) and 10% (6+60, DOSISPHERE) of patients could have participated in the study and, more importantly in the case of the SARAH trial, received radioembolization treatment with demonstrated improved tumor response and quality of life over the sorafenib treatment arm.

Recent studies have confirmed that the current treatment planning with ^99m^Tc‐MAA planar scintigraphy overestimates the actual ^90^Y‐microsphere LSF and LD and have demonstrated the improvement of ^99m^Tc‐MAA‐based LSF and LD accuracy and reproducibility by using SPECT/CT imaging instead of planar scintigraphy in both phantoms and patients.^6^, ^7^, ^8^, ^9^, ^10^, ^11^, ^12^ One publication, for example, found that SPECT‐based LSF and LD ($LSF^{\mathrm{spect}}$, $LD^{\mathrm{spect}}$) were on average 63% and 53% lower than the SOC $LSF^{\mathrm{planar}}$ and $LD^{\mathrm{planar}}$ values, respectively.^12^ Unfortunately, the over‐estimated SOC $LSF^{\mathrm{planar}}$ and $LD^{\mathrm{planar}}$ cannot be used to reliably predict the more accurate and lower $LSF^{\mathrm{spect}}$ and $LD^{\mathrm{spect}}$ values, as seen by the broad range of errors reported between the respective planar‐ and SPECT‐based values.^10^, ^11^, ^12^

While the accuracy improvement of SPECT/CT over planar imaging has been demonstrated in phantoms and patients, planar imaging remains prevalent as SOC for lung dosimetry and, in many clinical practices, the only possible imaging after ^99m^Tc‐MAA administration. However, the two sets of instructions outlined with varying amount of detail in the device IFUs are only two of the many possible ways of calculating $LSF^{\mathrm{planar}}$ and $LD^{\mathrm{planar}}$. The purpose of this work is to evaluate the accuracy and variability of various planar imaging algorithms for calculating $LSF^{\mathrm{planar}}$ and $LD^{\mathrm{planar}}$ at our institution.

This work does not seek to establish a de facto $LSF^{\mathrm{planar}}$ and $LD^{\mathrm{planar}}$ calculation to be implemented across all practices for all glass‐ and/or resin‐microsphere radioembolization patients. Instead, this retrospective analysis focuses on how different approaches in the dosimetry calculations from the same set of planar imaging inputs can change the predicted $LSF^{\mathrm{planar}}$ and $LD^{\mathrm{planar}}$. To this end, analysis of the patient cohort consisted of two components: first, determining which $LSF^{\mathrm{planar}}$ and $LD^{\mathrm{planar}}$ methodology resulted in values closest to $LSF^{\mathrm{spect}}$ and $LD^{\mathrm{spect}}$, and second, quantifying the relative impact of planar view, region‐of‐interest (ROI) contour, and liver shine‐through correction on $LSF^{\mathrm{planar}}$ accuracy.

## 2 MATERIALS AND METHODS

### 2.1 Patient cohort and imaging protocols

The cohort in this institutional review board‐approved retrospective analysis consisted of all consecutive patients with hepatocellular carcinoma (HCC) who underwent treatment planning for ^90^Y radioembolization with glass microspheres at our institution between 1 January 2015 and 31 December 2016. Forty‐six consecutive patients (9 women, 37 men) underwent 52 planning procedures (7 women once, 2 women twice, 33 men once, 4 men twice) and 39 patients (85%) underwent 44 treatment procedures (7 women once, 1 woman twice, 27 men once, 4 men twice). There were no strict patient selection criteria applied for data used in the study because the comparisons between LSF and LD were based on matched inputs between various approaches and therefore independent of the specifics of the patient population or disease status.

All LSF and LD values were derived from three imaging series acquired as part of SOC treatment planning: diagnostic chest CT, ^99m^Tc‐MAA planar scintigraphy, and ^99m^Tc‐MAA SPECT/CT. Planar (140 keV center and 15% width photopeak, 40 × 52 cm FOV, LEHR collimator, 7 min/view) and SPECT (140 keV center and 15% width photopeak, 15% width lower‐scatter window, 40 × 52 cm FOV, LEHR collimator, 16 seconds/view, 3D‐OSEM iterative reconstruction with attenuation, scatter, resolution recovery corrections, 5 mm FWHM Gaussian filter) imaging protocols followed previously published methodologies.^12^ Analysis of $LSF^{\mathrm{planar}}$ and $LD^{\mathrm{planar}}$ in this work was not differentiated based on patient sex, age, treatment volume (lobar or whole liver), or treatment number as each unique planning and treatment procedure served as its own control in all calculations.

### 2.2 ^99m^Tc‐MAA SPECT/CT dosimetry

Following the recommendations in previous publications,^10^, ^11^, ^12^ $LD^{\mathrm{spect}}$ was calculated using patient‐specific lung mass (*M* ^pat^) from densitovolumetry of a diagnostic chest CT, $LSF^{\mathrm{spect}}$ from liver and lung ^99m^Tc‐MAA SPECT counts, and the administered ^90^Y activity during the treatment procedure ($A_{90Y}$).

(1) $$\begin{array}{ccc} LD^{\mathrm{spect}}\;\left[Gy\right] & = & 49,670\;\left[\frac{Gy\cdot g}{GBq}\right]\times LSF^{\mathrm{spect}}\\ & & \times \;A_{90Y}\left[GBq\right]\times \frac{1}{M^{\mathrm{pat}}\left[g\right]} \end{array}$$

Left lung (*L*), right lung (*R*), and liver (*H*) SPECT counts were measured in contours manually drawn on the rigidly registered CT imaging using MIM Software. However, because of differences in spatial resolution and in respiratory and patient motion between SPECT and CT acquisitions, SPECT signal originating in the liver is often found outside of the registered CT liver boundaries. To account for this liver ^99m^Tc‐MAA signal recorded outside CT liver boundaries, the liver CT contour was morphologically expanded by 2 cm, and all SPECT counts within the expanded liver CT contour were attributed to the liver ($C_{H}^{\mathrm{spect}}$).

$LSF^{\mathrm{spect}}$ was calculated as the ratio of the estimated total lung counts ($C_{LR}^{\mathrm{spect}}$) to the estimated total liver counts (Equation 2).

(2) $$LSF^{\mathrm{spect}}\;=\frac{C_{LR}^{\mathrm{spect}}}{C_{H}^{\mathrm{spect}}}$$

However, to minimize the likelihood of liver ^99m^Tc‐MAA signal shine‐through effect inflating the SPECT counts within the CT lung boundaries, the total lung counts were estimated by first calculating the left lung SPECT counts ($C_{L}^{\mathrm{spect}}$) and the patient‐specific left lung mass ($M_{L}^{\mathrm{pat}}$) only within the left lung CT contour superior to the 2 cm liver CT expansion to estimate the average lung count density. Based on our previous work, the left lung is generally farther away from the superior liver and thus will be less contaminated by the liver shine through. The total lung counts ($C_{LR}^{\mathrm{spect}}$) were then calculated as the product of the average lung count density and the total lung mass from the diagnostic chest CT (Equation 3).

(3) $$C_{LR}^{\mathrm{spect}}\;=\frac{C_{L}^{\mathrm{spect}}}{M_{L}^{\mathrm{pat}}\left[g\right]}\times M^{\mathrm{pat}}\left[g\right]$$

By using the total lung mass from the diagnostic chest CT, this approach will also compensate for any signal from the apex of any lungs that may have been truncated in the SPECT/CT field of view.^12^

### 2.3 ^99m^Tc‐MAA planar dosimetry

This work investigated four factors involved in planar dosimetry: (1) planar view(s) selected, (2) ROI combination selected, (3) lung ROI count liver shine‐through correction applied, and (4) lung mass estimated. The first three factors dictate the lung and liver ROI counts used in the $LSF^{\mathrm{planar}}$ calculation (Equation 4), while the fourth factor is in the denominator of the $LD^{\mathrm{planar}}$ calculation (Equation 5). Like the $LD^{\mathrm{spect}}$ calculations above, $LD^{\mathrm{planar}}$ values were calculated using the actual administered ^90^Y activity during treatment ($A_{90Y}$).

(4) $$LSF^{\mathrm{planar}}=\frac{\left(\mathrm{Lung}\;\mathrm{counts}\right)}{\left(\mathrm{Lung}\;\mathrm{counts}\right)\;+\;\left(\mathrm{Liver}\;\mathrm{counts}\right)}$$

(5) $$\begin{array}{ccc} LD^{\mathrm{planar}}\left[\mathrm{Gy}\right] & = & 49,670\left[\frac{\mathrm{Gy}\cdot g}{\mathrm{GBq}}\right]\times \mathrm{LS}F^{\mathrm{planar}}\\ & & \times A_{90Y}\left[\mathrm{GBq}\right]\times \frac{1}{M\left[g\right]} \end{array}$$

**FIGURE 1:**
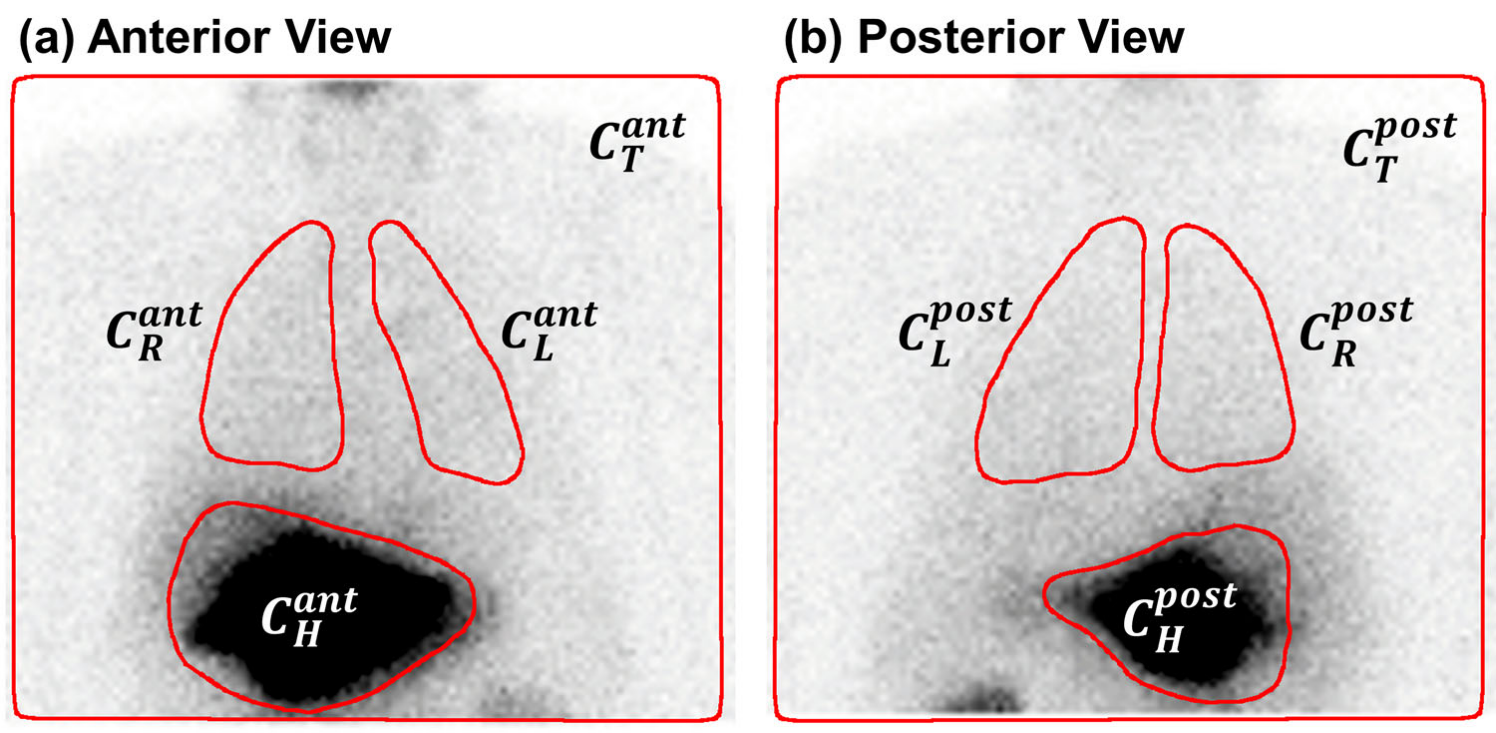
Example contours of the liver, left lung, right lung, and total frame in ^99m^Tc‐macro‐aggregated albumin (^99m^Tc‐MAA) anterior (a) and posterior (b) views of patient undergoing standard‐of‐care $LSF^{planar}$ calculations (Equations (6), (7), (8), (9), (10), (11)) for radioembolization treatment planning, illustrating the variability and uncertainty in delineating the organs of interest with freehand contours.

Left lung (*L*), right lung (*R*), and liver (*H*) ROIs were contoured free‐hand on anterior (ant) and posterior (post) planar scintigraphy views by trained nuclear medicine technologists (each with 3+ years of experience) and verified by nuclear medicine physician (with 12+ years of experience) to define the lung ($C_{LR}=C_{L}+C_{R}$) and liver ($C_{H}$) counts in each view (Figure 1). Additionally, the total measured counts ($C_{T}$) in each individual view were recorded for each patient.

### 2.4 Best planar dosimetry

The best planar dosimetry methodology in the study cohort was defined as the $LSF^{\mathrm{planar}}$ and $LD^{\mathrm{planar}}$ calculations ($LSF_{\mathrm{best}}^{\mathrm{planar}}$ and $LD_{\mathrm{best}}^{\mathrm{planar}}$, respectively) that most closely estimated the corresponding $LSF^{\mathrm{spect}}$ and $LD^{\mathrm{spect}}$ values. SPECT/CT‐based values were selected as the benchmark for comparison as they have been shown to most accurately quantify the true ^99m^Tc‐MAA distribution and therefore most accurately represent the post‐radioembolization ^90^Y lung shunt and dose. More specifically, $LSF_{\mathrm{best}}^{\mathrm{planar}}$ was defined as the $LSF^{\mathrm{planar}}$ methodology with the lowest median absolute difference (in pp) from $LSF^{\mathrm{spect}}$ across all patients undergoing treatment planning, while $LD_{\mathrm{best}}^{\mathrm{planar}}$was defined as the $LD^{\mathrm{planar}}$ methodology with the lowest median absolute difference (in Gy) relative to $LD^{\mathrm{spect}}$ across all patients undergoing treatment procedures.

#### 2.4.1 Best planar LSF

A total of 18 different $LSF^{\mathrm{planar}}$ methodologies were evaluated per planning procedure. For each planning procedure, six separate $LSF^{\mathrm{planar}}$ estimates (Equations (6), (7), (8), (9), (10), (11)) were initially calculated through the combination of three possible view choices (anterior only, posterior only, or geometric mean) and two possible contour choices [lungs and liver (LR,H); lungs and total frame (LR,T)] with no lung ROI count liver shine‐through correction.

(6) $$LSF_{LR,H}^{\mathrm{ant}}=\frac{C_{LR}^{\mathrm{ant}}}{C_{LR}^{\mathrm{ant}}\;+\;C_{H}^{\mathrm{ant}}}$$

(7) $$LSF_{LR,T}^{\mathrm{ant}}=\frac{C_{LR}^{\mathrm{ant}}}{C_{T}^{\mathrm{ant}}}$$

(8) $$LSF_{LR,H}^{\mathrm{post}}=\frac{C_{LR}^{\mathrm{post}}}{C_{LR}^{\mathrm{post}}\;+\;C_{H}^{\mathrm{post}}}$$

(9) $$LSF_{LR,T}^{\mathrm{post}}=\frac{C_{LR}^{\mathrm{post}}}{C_{T}^{\mathrm{post}}}$$

(10) $$\mathrm{LS}F_{\mathrm{LR},H}^{\mathrm{geo}}=\frac{\sqrt{C_{\mathrm{LR}}^{\mathrm{ant}}\times C_{\mathrm{LR}}^{\mathrm{pos}t}}}{\sqrt{C_{\mathrm{LR}}^{\mathrm{ant}}\times C_{\mathrm{LR}}^{\mathrm{pos}t}}+\sqrt{C_{H}^{\mathrm{ant}}\times C_{H}^{\mathrm{pos}t}}}$$

(11) $$\mathrm{LS}F_{\mathrm{LR},T}^{\mathrm{geo}}=\frac{\sqrt{C_{\mathrm{LR}}^{\mathrm{ant}}\times C_{\mathrm{LR}}^{\mathrm{pos}t}}}{\sqrt{C_{T}^{\mathrm{ant}}\times C_{T}^{\mathrm{pos}t}}}$$

For each of the six view and contour combinations, two additional $LSF^{\mathrm{planar}}$ values were calculated to test two simple liver shine‐through corrections of the lung ROI counts, resulting in a total of 18 different $LSF^{\mathrm{planar}}$ calculations. Based on previous work developing a SPECT/CT methodology noting that the right lung counts were primarily affected by liver shine‐through effects, the measured right lung counts $C_{R}$ in each of the Equations (6)–(11) was replaced with “liver‐shine‐through‐free” right lung counts estimated in one of the two ways. In the first, the corrected right lung counts were calculated as $C_{Rc}=1.15\;\times C_{L}$, where the constant 1.15 factor corresponds to the standard man assumption that the right lung is 15% larger than the left lung.^20^ In the second, the corrected right lung counts were calculated as $C_{Rc}=\left(A_{R}/A_{L}\right)\;\times C_{L}$, where $A_{R}/A_{L}$ corresponds to the ratio of the drawn right lung to left lung ROI areas. Both of these corrections assume the lungs have similar perfusion and the left lung ROI counts are minimally impacted by liver‐originating ^99m^Tc‐MAA signal.^12^

The median and range of calculated $LSF^{\mathrm{planar}}$ values (in pp) and of pairwise differences between $LSF^{\mathrm{spect}}$ and each of the 18 $LSF^{\mathrm{planar}}$ methodologies across all planning procedures is reported. $LSF_{\mathrm{best}}^{\mathrm{planar}}$ was defined as the methodology with the lowest median absolute difference.

#### 2.4.2 Best planar lung dose

A total of four different $LD^{\mathrm{planar}}$ methodologies were calculated from the combination of two different $LSF^{\mathrm{planar}}$ values and two different lung mass values. The two $LSF^{\mathrm{planar}}$ values were either the SOC methodology at our institution of $LSF_{LR,H}^{\mathrm{geo}}$ (Equation 10) or the $LSF_{\mathrm{best}}^{\mathrm{planar}}$ determined above, while the two lung mass values were either the standard 1,000 g lung mass IFU assumption (*M* ^std^) or the patient‐specific *M* ^pat^ derived from diagnostic chest CTs used in the $LD^{\mathrm{spect}}$ calculation. In each treatment procedure, all four $LD^{\mathrm{planar}}$ calculations (Equation 5) were made using the same actual administered ^90^Y activity, where the net administered activity was calculated per the TheraSphere IFU.^2^ The median and range of calculated doses (Gy) and of pairwise differences between $LD^{\mathrm{spect}}$ and each of the four $LD^{\mathrm{planar}}$ methodologies across all treatment procedures are reported.

## 3 RESULTS

### 3.1 Best planar dosimetry

**FIGURE 2:**
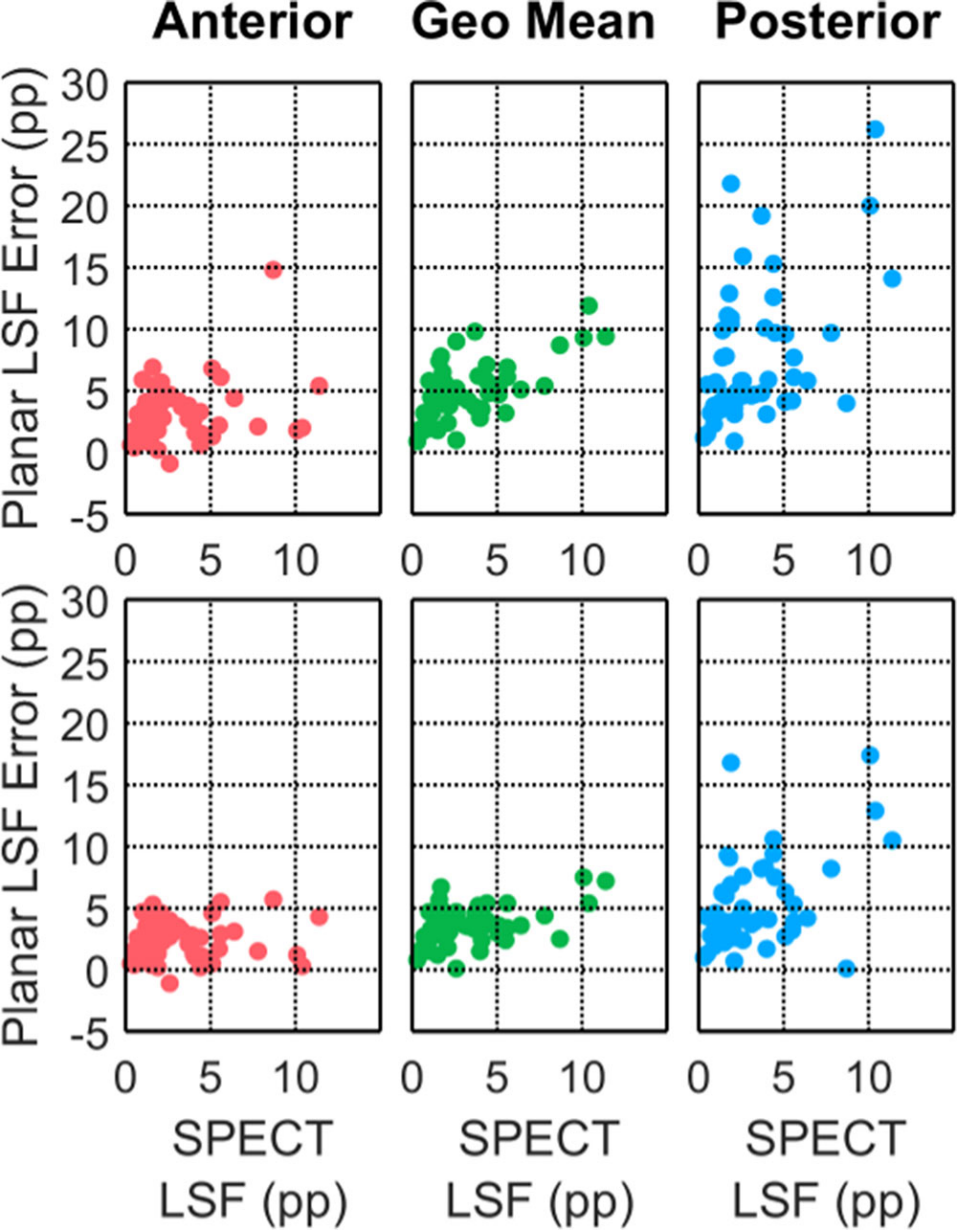
Bland–Altman of absolute $LSF^{\mathrm{planar}}$ errors = $LSF^{\mathrm{planar}}-LSF^{\mathrm{spect}}\;$(percentage points, pp) in 55 planning procedures using the anterior, geometric mean, and posterior views and the lung and liver contours ($LSF_{LR,H}$, top row) and lung and total frame contours ($LSF_{LR,T}$, bottom row).

**FIGURE 3:**
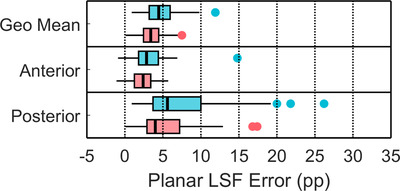
Box‐and‐whisker plots of absolute $LSF^{\mathrm{planar}}$ errors = $LSF^{\mathrm{planar}}-LSF^{\mathrm{spect}}\;$(percentage points, pp) in 55 planning procedures for 6 different $LSF^{\mathrm{planar}}$ methodologies with no lung region‐of‐interest (ROI) count liver shine‐through correction. For each view selection, $LSF^{\mathrm{planar}}$ errors from lung and liver contour selection ($LSF_{LR,H}$) and from lung and total frame contour selection ($LSF_{LR,T}$) are shown in blue and red, respectively.

In the 52 planning procedures, the median (range) $LSF^{\mathrm{spect}}$ was 2 pp (0–11 pp). Table 1 shows the absolute errors for the 18 possible $LSF^{\mathrm{planar}}$ calculations. Figures 2 and 3, respectively, show the Bland–Altman and boxplots of absolute errors for the six possible $LSF^{\mathrm{planar}}$ calculations without shine‐through corrections. Based on these results, $LSF_{\mathrm{best}}^{\mathrm{planar}}$ was $LSF_{LR,T}^{\mathrm{ant}}$ without shine‐through corrections with a median (maximum) error of 2.4 pp (5.7 pp). By contrast, the SOC $LSF_{LR,H}^{\mathrm{geo}}$ without shine‐through corrections had median (maximum) error of 4.4 pp (11.9 pp).

**TABLE 1:** Distribution of median (minimum, maximum) absolute $LSF^{\mathrm{planar}}$ errors = $LSF^{\mathrm{planar}}-LSF^{\mathrm{spect}}\;$(percentage points, pp) in 55 planning procedures for 18 different $LSF^{\mathrm{planar}}$ methodologies.

		**Lung counts calculation**		
**View**	**Contours**	$C_{L}+C_{R}$	$C_{L}+1.15C_{L}$	$C_{L}+\left(A_{R}/A_{L}\right)C_{L}$
***Geometric mean***	***Lungs, liver***	4.4 (0.9, 11.9) pp	3.8 (0.3, 14.1) pp	3.7 (0.3, 16.0) pp
	***Lungs, total***	3.4 (0.1, 7.5) pp	2.7 (−0.4, 12.9) pp	3.9 (0.3, 25.3) pp
***Anterior***	***Lungs, liver***	2.8 (−0.9, 14.8) pp	2.1 (−1.6, 14.1) pp	1.9 (−1.1, 11.2) pp
	***Lungs, total***	2.4 (−1.1, 5.7) pp	1.4 (−1.7, 6.2) pp	2.0 (−1.0, 16.1) pp
***Posterior***	***Lungs, liver***	5.6 (0.9, 26.2) pp	5.1 (0.7, 30.2) pp	5.0 (0.5, 31.8) pp
	***Lungs, total***	4.0 (0.1, 17.4) pp	3.6 (0.5, 27.0) pp	5.7 (0.6, 62.7) pp

Patient‐specific lung mass (*M* ^pat^) from densitovolumetry of diagnostic chest CT yielded median (range) values of 816 g (548–1178 g). Stated otherwise, the 1,000 g *M* ^std^ assumption over‐estimated *M* ^pat^ lung mass by a median (range) 22% (−15% to 82%) in this cohort.

**FIGURE 4:**
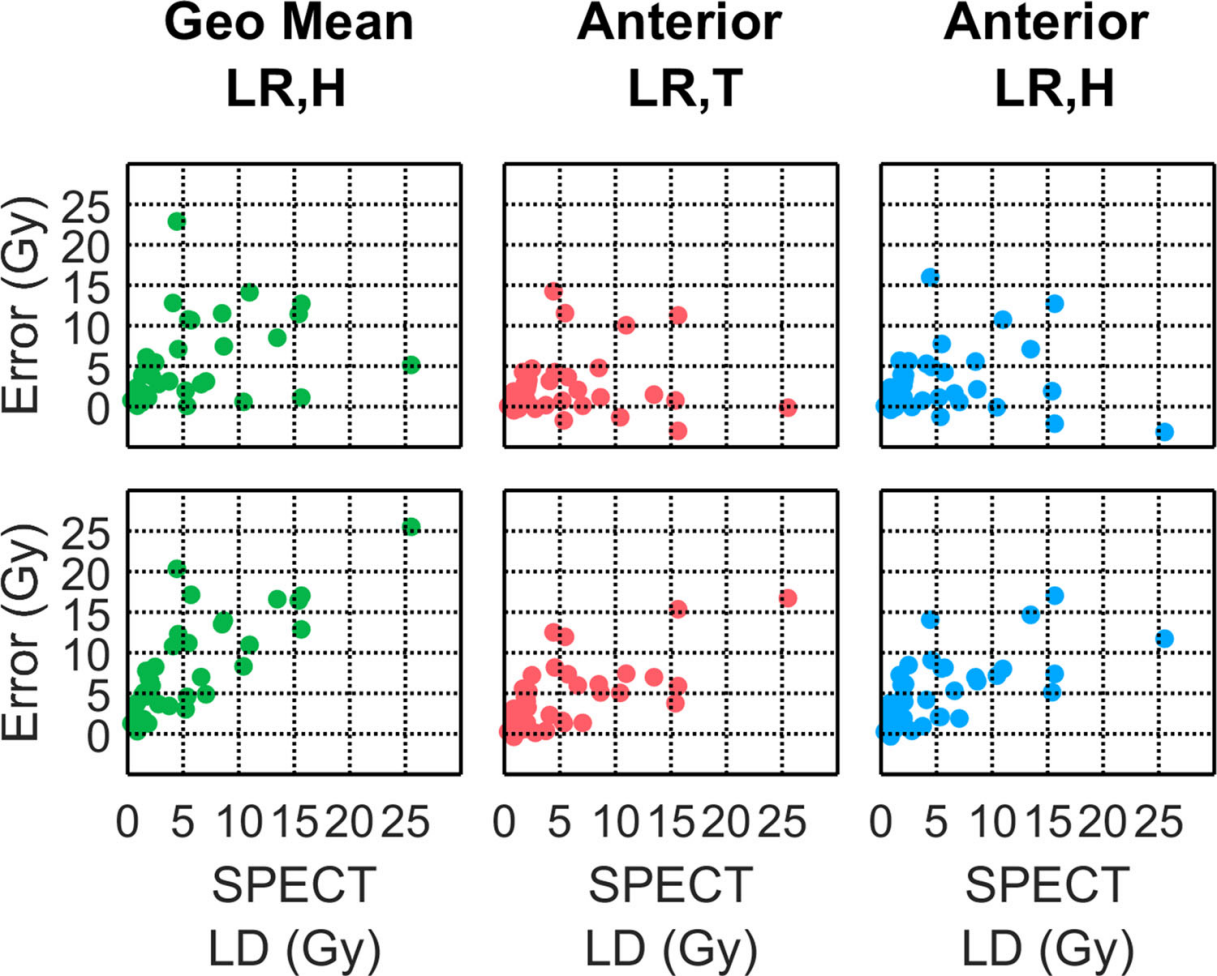
Bland–Altman of absolute $LD^{\mathrm{planar}}$ errors = $LD^{\mathrm{planar}}-LD^{\mathrm{spect}}\;$(Gy) in 44 treatment procedures using $LSF_{LR,H}^{\mathrm{geo}},\;LSF_{LR,T}^{\mathrm{ant}}$, and $LSF_{LR,H}^{\mathrm{ant}}\;$with either 1 kg (top row) or patient‐specific (bottom row) lung masses.

**FIGURE 5:**
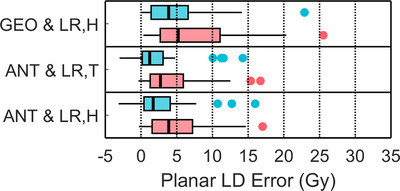
Box‐and‐whisker plots of absolute $LD^{\mathrm{planar}}$ errors = $LD^{\mathrm{planar}}-LD^{\mathrm{spect}}\;$(Gy) in 44 planning procedures for six different $LD^{\mathrm{planar}}$ methodologies with no lung region‐of‐interest (ROI) count liver shine‐through correction. For each $LSF^{\mathrm{planar}}$ selection, $LD^{\mathrm{planar}}$ errors from 1 kg lung mass (*M* ^std^) and from patient‐specific lung mass (*M* ^pat^) are shown in blue and red, respectively.

In the 52 treatment procedures, the median (range) $LD^{\mathrm{spect}}$ was 2.1 Gy (0.3–25.5 Gy). Table 2 summarizes the distribution of calculated doses and calculated differences relative to $LD^{\mathrm{spect}}$ for each of the $LD^{\mathrm{planar}}$ calculations. Figures 4 and 5, respectively, show the Bland–Altman and boxplots of the absolute errors reported in Table 2. Based on these results, $LD_{\mathrm{best}}^{\mathrm{planar}}$ was $LD_{std}^{\mathrm{ant}|LR,T}$ (i.e., using the best $LSF_{LR,T}^{\mathrm{ant}}$ and 1,000 g standard lung mass) with lowest median absolute errors from $LD^{\mathrm{spect}}$ of 1.2 Gy (error range of −3.0 to 14.3 Gy). By contrast, the SOC $LD_{\mathrm{std}}^{\mathrm{geo}|LR,H}$ (i.e., using $LSF_{LR,H}^{\mathrm{geo}}$ and *M* ^std^) median (range) had a median (range) absolute error of 3.9 Gy (0.1–22.9 Gy).

**TABLE 2:** Distribution of median (minimum, maximum) calculated doses (Gy) and absolute errors ($LD^{\mathrm{planar}}-LD^{\mathrm{spect}}$) in 44 treatment procedures for six different $LD^{\mathrm{planar}}$ methodologies.

**Planar LD methodology**			**Dose (Gy)**		**Error (Gy)**	
**LSF view**	**LSF contours**	**Lung mass**	**Median**	**Range**	**Median**	**Range**
**Geometric mean**	**Lungs, liver**	**1** **kg**	6.4	(1.0, 31)	3.9	(0.1, 23)
		**CT based**	8.4	(1.2, 51)	5.2	(0.3, 26)
**Anterior**	**Lungs, total**	**1** **kg**	4.7	(0.4, 27)	1.2	(−3.0, 14]
		**CT based**	6.4	(0.5, 42)	2.7	(−0.4, 17)
**Anterior**	**Lungs, liver**	**1** **kg**	5.4	(0.4 28)	1.7	(−3.1, 16)
		**CT based**	7.4	(0.6, 37)	3.9	(−0.3, 17)

## 4 DISCUSSION

The clinical practice of ^90^Y‐microsphere radioembolization has evolved in recent years to incorporate advanced 3D functional and anatomical imaging to improve treatment planning and dosimetry. Lung dosimetry with 3D functional/anatomical imaging has been demonstrated to provide the most accurate and reproducible values.^6^, ^7^, ^8^, ^9^, ^10^, ^11^, ^12^ In practice, however, 2D imaging remains the SOC for lung dosimetry, so it is important to understand how the biases and errors in 2D lung dosimetry can negatively impact patient management.

From first principles, the most accurate planar lung dosimetry was expected to be estimated using $LSF^{\mathrm{planar}}$ from anterior view lung and liver contours, and patient‐specific lung masses. However, in this work, the ^99m^Tc‐MAA‐based $LSF^{\mathrm{planar}}$ methodology resulting in the closest values to ^99m^Tc‐MAA‐based $LSF^{\mathrm{spect}}$ values were calculated as the ratio of lung counts to total image counts in the anterior view of the planar image (i.e., $LSF_{LR,T}^{\mathrm{ant}}$). Compared to the SOC $LSF_{LR,H}^{\mathrm{geo}}$ methodology using the ratio of the geometric mean of lung counts to the geometric mean of liver counts, the more accurate $LSF_{LR,T}^{\mathrm{ant}}$ methodology reduced the median (maximum) LSF over‐estimation from 5 pp (12 pp) to 2 pp (6 pp). Although a 1,000 g lung mass generally over‐estimated patient‐specific lung masses, the closest $LD^{\mathrm{planar}}$ values to $LD^{\mathrm{spect}}$ were calculated using $LSF_{LR,T}^{\mathrm{ant}}$ and the 1,000 g lung mass assumption.

### 4.1 Planar LSF bias from view selection

The geometric mean $LSF^{\mathrm{planar}}$ calculation, as outlined in the IFUs, does not account for the differences in scatter and attenuation coefficients between the patient's abdomen and chest and therefore does not correct for preferential attenuation between views. Namely, both the liver and lung ROI count estimations lack their respective $e^{\mu x}$ attenuation correction, where μ is the effective linear attenuation coefficient and *x* is the body thickness.^21^ Because the liver is more attenuating than the lungs, applying the $e^{\mu x}$ factor would increase the “corrected” liver counts more than the “corrected” lung counts, resulting in a lower “corrected” geometric mean $LSF^{\mathrm{planar}}$ than the IFU $LSF^{\mathrm{planar}}$ (Equation 4).

In general, using only the anterior view data resulted in the best $LSF^{\mathrm{planar}}$ accuracy. Incidentally, the liver is located preferentially anterior within the torso. As a result, photons emitted from the liver will be attenuated to a greater extent on their longer path toward the posterior detector than toward the anterior detector. Therefore, from first principles, the anterior view will typically contain higher liver counts relative to the posterior view.

Allred et al. overcome the inaccuracies introduced by the preferential signal attenuation by the liver and heart by calculating lung shunt using only the liver ROI counts in the anterior view and the lung ROI counts in the posterior view.^8^ In a torso phantom, they report that this approach reduced the LSF overestimation in the SOC geometric mean calculation from up to ∼6 pp down to ∼2 pp across a range of LSFs < 10 pp. Although this exact methodology was not evaluated in this study, our results corroborate their conclusion that using data from a single view leads to more accurate lung dosimetry than applying geometric mean calculations on data from two views.

**FIGURE 6:**
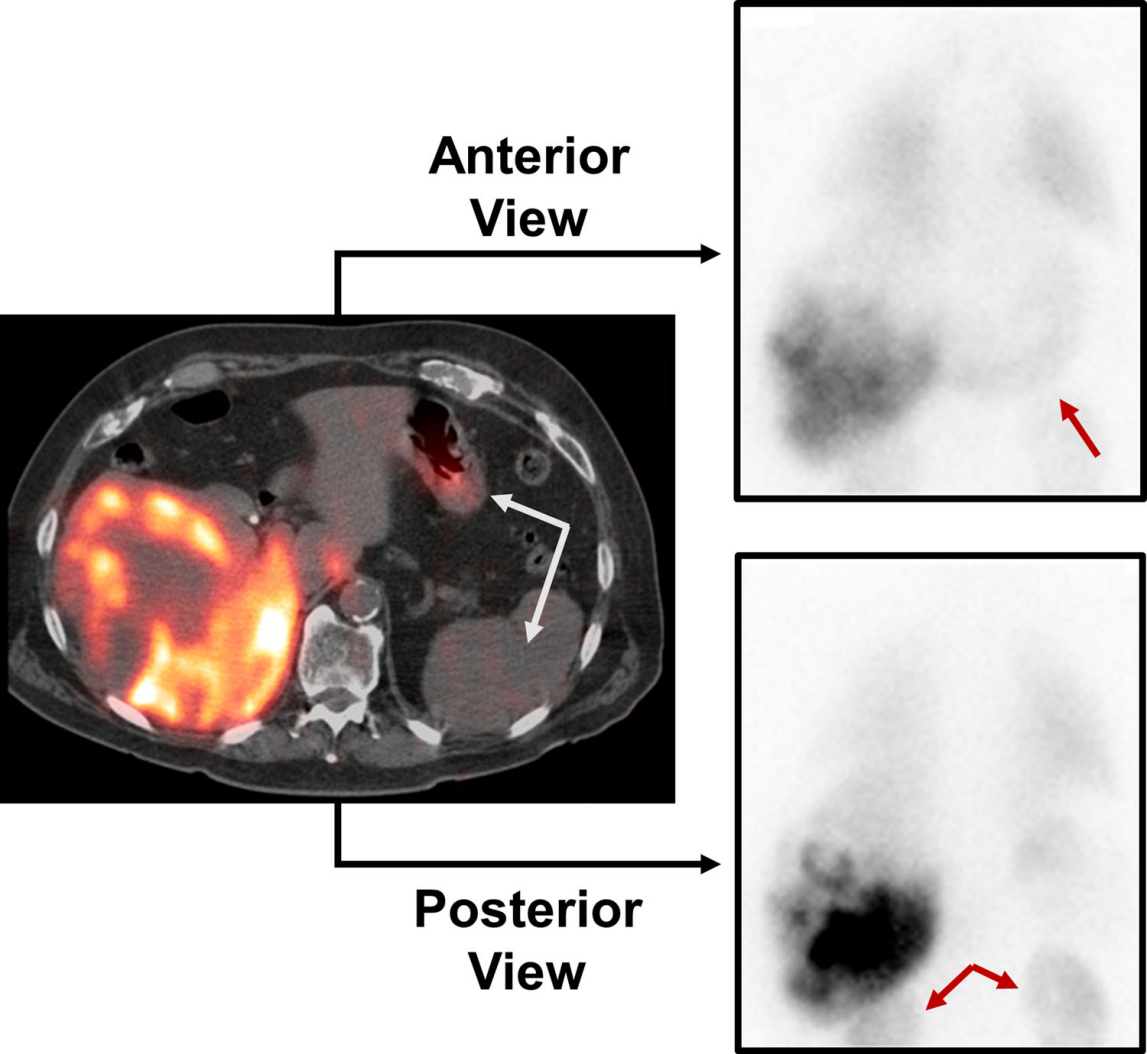
Example patient (Patient D in Table 3) with $LSF^{\mathrm{spect}}$ of 0.09 with primarily posterior distribution of ^99m^Tc‐macro‐aggregated albumin (^99m^Tc‐MAA) within the liver, as seen in the fused SPECT/CT axial slice. As a result, the posterior view exhibited higher liver intensity (both planar images displayed using same window width/level) and resulted in the more accurate 0.13 $LSF_{LR,H}^{\mathrm{post}}$ than the anterior view 0.24 $LSF_{LR,H}^{\mathrm{ant}}$. When calculating $LSF^{\mathrm{planar}}$ using the total image counts, the additional signal from the bowel and kidneys (denoted in arrows) decreases values to 0.09 and 0.14 for $LSF_{LR,T}^{\mathrm{post}}$ and $LSF_{LR,T}^{\mathrm{ant}}$, respectively.

$LSF^{\mathrm{ant}}$ was more accurate than $LSF^{\mathrm{post}}$ in 92% of planning procedures (48/52) regardless of contour choice. In the four (8%) planning procedures with more accurate $LSF^{\mathrm{post}}$, $LSF^{\mathrm{ant}}$ methodologies without liver shine‐through corrections differed anywhere between 4 and 15 pp from $LSF^{\mathrm{spect}}$ whereas $LSF^{\mathrm{post}}$ methodologies only differed by 1 pp to 5 pp from $LSF^{\mathrm{spect}}$ (Table 3). In the worst case scenario with Patient D, $LSF_{LR,H}^{\mathrm{ant}}$ of 24 pp was 170% higher than the assumed true $LSF^{\mathrm{spect}}$ of 9 pp but only 40% higher than the more accurate $LSF_{LR,H}^{\mathrm{post}}$ of 13 pp (Figure 6). Upon further inspection, all four cases had primarily posteriorly distributed ^99m^Tc‐MAA on their fused SPECT/CT imaging, resulting in higher posterior than anterior planar liver counts and thus lower and more accurate posterior view‐based LSFs. These results indicate that the lower value between $LSF^{\mathrm{ant}}$ and $LSF^{\mathrm{post}}$ will be the more accurate $LSF^{\mathrm{planar}}$ value for treatment planning. Thus, we conclude that planar lung dosimetry accuracy was improved in our patient cohort by not using $LSF^{\mathrm{geo}}$ approaches but instead by selecting the most appropriate single view for quantitation after reviewing both $LSF^{\mathrm{ant}}$ and $LSF^{\mathrm{post}}$ values alongside patient imaging.

**TABLE 3:** $LSF^{\mathrm{post}}$ and $LSF^{\mathrm{ant}}$ in patients with more accurate $LSF^{\mathrm{post}}$ values (percentage points, pp).

	$LSF^{\mathrm{spect}}\;\left(\mathrm{pp}\right)$	$LSF_{LR,T}^{\mathrm{post}}\;\left(\mathrm{pp}\right)$	$LSF_{LR,H}^{\mathrm{post}}\;\left(\mathrm{pp}\right)$	$LSF_{LR,T}^{\mathrm{ant}}$ **(pp)**	$LSF_{LR,H}^{\mathrm{ant}}\;\left(\mathrm{pp}\right)$
**Patient A**	2	3	3	6	6
**Patient B**	2	5	5	7	8
**Patient C**	5	8	9	10	12
**Patient D**	9	9	13	14	24

### 4.2 Planar LSF bias from contour selection

Contouring is one of the largest sources of uncertainty in calculating $LSF^{\mathrm{planar}}$ because the true lung and liver boundaries are hard to visualize and accurately delineate. Not only do the IFUs differ in whether or not to contour the lungs and liver separately, they do not provide a precise methodology to perform their respective contouring. For example, some practices acquire a separate scan with a ^99m^Tc flood source under the patient to guide with the lung contouring; some adjust the window width and level based on the maximum pixel value to guide liver contouring; some use the same contours for both anterior and posterior views; and some freehand everything. As a result, different institutions and even different individuals at the same institution may estimate different $LSF^{\mathrm{planar}}$ values for the same patient.

Our results indicate that $LSF_{LR,T}$ values were always equally close, if not closer, to $LSF^{\mathrm{spect}}$ than $LSF_{LR,H}$ values. However, this seeming improvement in accuracy is largely driven by the inclusion of excess extra‐hepatic and extra‐pulmonary ^99m^Tc‐signal in the denominator, that originates, not from ^99m^Tc‐MAA biodistribution, but rather from ^99m^Tc‐pertechnetate following the dissociation of ^99m^Tc‐MAA.^4^, ^5^ The additional ^99m^Tc signal can be observed in organs with typical intravenous ^99m^Tc‐pertechnetate uptake, such as the kidneys, thyroid, and stomach. In Patient D, for example, ^99m^Tc signal originating from the kidneys and stomach is especially evident in the posterior view (Figure 6) which lead to lower $LSF_{LR,T}$ versus $LSF_{LR,H}$ values (Table 3). As a consequence, the higher denominator in $LSF_{LR,T}$ versus $LSF_{LR,H}$ equations for the same numerator lowers the $LSF^{\mathrm{planar}}$ estimate and minimizes the known $LSF^{\mathrm{spect}}$ overestimation of all $LSF^{\mathrm{planar}}$ calculations. However, we found minimal differences in $LSF^{\mathrm{planar}}$ with contouring choice for the majority of cases (Table 1 and Figure 2), highlighting the low impact of extra‐hepatic signal on planar dosimetry accuracy. Therefore, practices can select which contouring choice works best for their treatment planning workflow as long as they are consistent for all patients and that they continue to review both anterior and posterior planar images for gross extra‐hepatic ^99m^Tc signal outside the expected MAA and pertechnetate biodistributions.

### 4.3 Planar LD biases: LSF versus lung mass estimations

$LD^{\mathrm{planar}}$ accuracy improved when the accuracy of $LSF^{\mathrm{planar}}$ improved (by using the appropriate $LSF^{\mathrm{ant}}$ or $LSF^{\mathrm{post}}$ view instead of $LSF^{\mathrm{geo}}$), but worsened when the accuracy of lung mass was improved (by using patient‐specific lung masses *M* ^pat^ instead of 1,000 g *M* ^std^). Based on Equation (5), $LD^{\mathrm{planar}}$ values will approach the lower, more accurate, $LD^{\mathrm{spect}}$ values as the ratio of $LSF^{\mathrm{planar}}$ to the estimated lung mass (*M* ^est^) approaches the ratio of $LSF^{\mathrm{spect}}$ to *M* ^pat^. So, while anatomically incorrect, the 1000 g *M* ^std^ assumption, which, on average, over‐estimates the true lung mass *M* ^pat^, results in more accurate $LD^{\mathrm{planar}}$ estimates than using *M* ^pat^.

To date, there is no established guidance on using patient‐specific lung masses for treatment planning nor are there updated lung shunt and dose limits based on the more accurate and personalized measurements possible with 3D functional and anatomical imaging. Therefore, at this time, the value of calculating patient‐specific lung mass in planar dosimetry is unknown, and at least within our patient cohort, the additional burden of this calculation is not even warranted. Nevertheless, practices should beware the possible discrepancies of calculated LDs with 1,000 g versus patient‐specific lung masses, especially in patients with smaller lungs.

### 4.4 Limitations and future directions

Shortcomings of the work include the use of clinical data from a single institution and the limited range of lung shunts observed in the patient population. Of the 52 planning procedures, 28 (54%,) all had an $LSF^{\mathrm{planar}}$ < 10 pp regardless of planar views, contours, or liver shine‐through correction combination calculated. Finally, all calculations in this work are based on imaging ^99m^Tc‐MAA biodistribution, which does not always reflect the eventual ^90^Y‐microsphere biodistribution, especially in the lung compartment. The impact of scanner type, ^99m^Tc‐MAA activity, image acquisition time, and inter‐reader contour variability and other factors on $LSF^{\mathrm{planar}}$ and $LD^{\mathrm{planar}}$ accuracy and precision were not investigated. The variability in contouring and the relatively low lung signal are likely the major reasons why our liver shine‐through correction attempts did not improve $LSF^{\mathrm{planar}}$ accuracy as they have $LSF^{\mathrm{spect}}$ accuracy.

Nevertheless, the concepts presented in this work could be used in clinical practice to improve our protocols for ^90^Y‐microsphere treatment planning with ^99m^Tc‐MAA. The fact that low $LSF^{\mathrm{geo}}$ values (<10%) are typically observed, along with the scarce evidence for radiation pneumonitis above the current SOC LD limits, have led to the proposition of an algorithm whereby high $LSF^{\mathrm{planar}}$ or high $LD^{\mathrm{planar}}$ alone are not used as a contraindication for radioembolization.^13^ Rather, patients with $LD^{\mathrm{planar}}$ expected to exceed 20 Gy would be assessed for risk of radiation pneumonitis by calculating $LD^{\mathrm{spect}}$ and consideration of other comorbidities.^13^ Future work is necessary to optimize the algorithm and establish new safety thresholds for the updated, more accurate, $LD^{\mathrm{planar}}$ and $LD^{\mathrm{spect}}$ calculations.

## 5 CONCLUSIONS

In this retrospective study, ^99m^Tc‐MAA‐based lung dosimetry accuracy with 2D planar imaging: (1) improved by only using a single view to calculate LSF instead of applying a geometric mean between anterior and posterior views, (2) improved slightly when using lung and total image contours to calculate LSF because of extra‐hepatic ^99m^Tc‐MAA signal, and (3) worsened when using patient‐specific lung masses to calculate dose instead of assuming 1,000 g lungs for all patients.

## CONFLICT OF INTEREST

Armeen Mahvash serves as a consultant for Boston Scientific, Sirtex Medical, and has research contracts with Boston Scientific, Sirtex Medical. Marnix G. E. H. Lam serves as a consultant for Boston Scientific, Terumo Medical, and has research contracts with Boston Scientific, Terumo Medical. S. Cheenu Kappadath serves as a consultant for Terumo Medical and has research contracts with ABK Biomedical, Boston Scientific, Sirtex Medical.

## AUTHOR CONTRIBUTIONS

Benjamin P. Lopez and S. Cheenu Kappadath were responsible for study design, data collection and analysis, and manuscript preparation. James P. Long assisted in statistical analysis. Armeen Mahvash and Marnix G. E. H. Lam provided clinical feedback. All authors read and approved the final manuscript.
