# Erratum: “Factors modulating ^99m^Tc‐MAA planar lung dosimetry for ^90^Y radioembolization”

**DOI:** 10.1002/acm2.14017

**Published:** 2023-06-06

Lopez, BP, Mahvash, A, Long, JP, Lam, MGEH, Kappadath, SC. Factors modulating ^99m^Tc‐MAA planar lung dosimetry for ^90^Y radioembolization. *J Appl Clin Med Phys*. 2022;23:e13734.

In Materials and Methods Section 2.2 | ^99m^Tc‐MAA SPECT/CT dosimetry (Page 3 of 11), the following text and equation in Paragraph 3 was incorrectly stated:

$LSF^{spect}$ was calculated as the ratio of the estimated total lung counts ($C_{LR}^{spect}$) to the estimated total liver counts (Equation 2).

(2) $$LS\;F^{spect}=\frac{C_{LR}^{spect}}{C_{H}^{spect}}\;$$

The correct text and equation describing the SPECT/CT‐based lung shunt fraction ($LSF^{spect}$) calculation should be:

$LSF^{spect}$ was calculated as the ratio of the estimated total lung counts ($C_{LR}^{spect}$) to the sum of the estimated total lung counts ($C_{LR}^{spect}$) and total liver counts ($C_{H}^{spect}$) (Equation 2).

(2) $$LS\;F^{spect}=\frac{C_{LR}^{spect}}{C_{LR}^{spect}+C_{H}^{spect}}\;$$

We have verified that the data analysis was based on the correct equation stated above. The published results and conclusions of the paper are valid. We apologize for this error.

